# A pre-intervention study of malaria vector abundance in Rio Muni, Equatorial Guinea: Their role in malaria transmission and the incidence of insecticide resistance alleles

**DOI:** 10.1186/1475-2875-7-194

**Published:** 2008-09-29

**Authors:** Frances C Ridl, Chris Bass, Miguel Torrez, Dayanandan Govender, Varsha Ramdeen, Lee Yellot, Amado Edjang Edu, Christopher Schwabe, Peter Mohloai, Rajendra Maharaj, Immo Kleinschmidt

**Affiliations:** 1 Malaria Research Lead Programme, Medical Research Council, 491 Ridge Road, Durban, South Africa; 2 Department of Biological Chemistry, Rothamsted Research, Harpenden, AL5 2JQ, UK; 3 Equatorial Guinea Malaria Control Initiative, Apdo # 606, Bata, Equatorial Guinea; 4 C/O.U.A., Zona Sanitaria s/n, Bata-Litoral, Equatorial Guinea; 5 Medical Care Development International, 8401 Colesville Rd, Silver Spring, Maryland, 20910, USA; 6 One World Development Group International, Punta Europa, Carretera Aeropuerto, Malabo, Bioco Norte, Equatorial Guinea; 7 London School of Hygiene and Tropical Medicine, Keppel St, London, WC1E 7HT, UK

## Abstract

**Background:**

Following the success of the malaria control intervention on the island of Bioko, malaria control by the use of indoor residual spraying (IRS) and long-lasting insecticide-treated nets (LLITN) was extended to Rio Muni, on the mainland part of Equatorial Guinea. This manuscript reports on the malaria vectors present and the incidence of insecticide resistant alleles prior to the onset of the programme.

**Methods:**

*Anopheles *mosquitoes were captured daily using window traps at 30 sentinel sites in Rio Muni, from December 2006 to July 2007. The mosquitoes were identified to species and their sporozoite rates, knockdown resistance *(kdr) *and acetylcholinesterase (AChE) sensitivity measured, to define the role of vector species in malaria transmission and their potential susceptibility to insecticides.

**Results:**

A total of 6,162 *Anopheles *mosquitoes were collected of which 4,808 were morphologically identified as *Anopheles gambiae s.l*., 120 *Anopheles funestus*, 1,069 *Anopheles moucheti*, and 165 *Anopheles nili s.l.*. Both M and S molecular forms of *Anopheles gambiae s.s*. and *Anopheles melas *were identified. *Anopheles ovengensis *and *Anopheles carnevalei *were the only two members of the *An. nili *group to be identified. Using the species-specific sporozoite rates and the average number of mosquitoes per night, the number of infective mosquitoes per trap per 100 nights for each species complex was calculated as a measure of transmission risk. Both *kdr-w *and *kdr-e *alleles were present in the S-form of *An. gambiae s.s. *(59% and 19% respectively) and at much lower frequencies in the M-form (9.7% and 1.8% respectively). The *kdr-w *and *kdr-e *alleles co-occurred in 103 S-form and 1 M-form specimens. No insensitive AChE was detected.

**Conclusion:**

*Anopheles gambiae s.s*, a member of the *Anopheles gambiae *complex was shown to be the major vector in Rio Muni with the other three groups playing a relatively minor role in transmission. The demonstration of a high frequency of *kdr *alleles in mosquito populations before the onset of a malaria control programme shows that continuous entomological surveillance including resistance monitoring will be of critical importance to ensure the chosen insecticide remains effective.

## Background

Malaria is a major endemic disease in Rio Muni, the mainland part of Equatorial Guinea situated at 1.512°N 10.267° on the west coast of Central Africa. Estimates from a *Plasmodium falciparum *prevalence survey conducted in 2007 among children between two and 15 years of age showed site-specific parasitaemias to vary from 54% to 89% with an average of 72% (unpublished data I. Kleinschmidt and L. Benavente). Following the success of the Bioko Island Malaria Control Project (BIMCP) [1,2] malaria control has been extended to Rio Muni under the Equatorial Guinea Malaria Control Initiative (EGMCI) by a staged roll-out of indoor residual house spraying (IRS) in Litoral and Kie-Ntem provinces and long-lasting insecticide-treated net (LLITN) distribution in the other two provinces (Cento Sur and Wele Nzas). Extensive information and education campaigns are being conducted and all areas will benefit from the introduction of free artemisinin-based combination therapy starting in July 2008. This initiative, to substantially reduce malaria on the mainland using IRS and LLITNs is being funded by the Global Fund to fight Aids, Tuberculosis and Malaria (GFATM) and Marathon Oil Company and is run in partnership with the government of Equatorial Guinea, Medical Care Development International (MCDI), One World Development Group International (OWDGI), Medical Research Council of South Africa (MRC), Harvard and Yale Universities and the London School of Hygiene and Tropical Medicine.

The tropical all year round humid climate and the many rivers and streams, both fast and slow flowing, provide ideal breeding conditions for different malaria vectors. Earlier studies have shown *Anopheles gambiae sensu lato (s.l.) *and *Anopheles funestus *to be the major vectors of malaria on the mainland of Equatorial Guinea [3-5]. This paper reports on the composition, density, infectivity, knockdown resistance (*kdr*) and insensitive acetylcholinesterase (iAChE) status of malaria vector species exiting houses through window traps before the start of the intervention.

## Materials and methods

### Entomological monitoring

**Figure 1:**
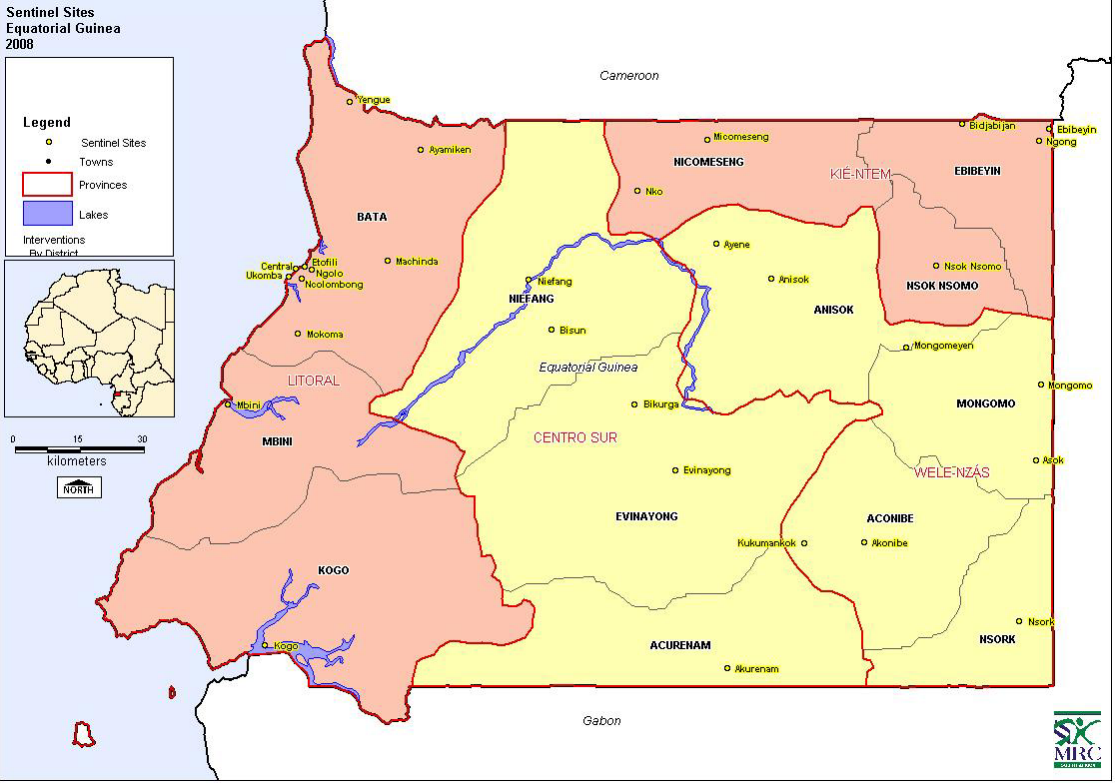
Map of the sentinel sites in the four provinces of Rio Muni, Equatorial Guinea.

In November 2006, window traps were installed at six houses at each of 30 sentinel sites selected in each of the four provinces (Figure 1); eleven sites in the coastal province, Litoral, which includes Bata, the principle city on the mainland, five sites in Centro Sur which comprises the centre of the mainland, six sites in Kie Ntem in the north-east and eight sites in Welas Nzas in the south-east. The extensive distribution of the sentinel sites throughout the country facilitated localized monitoring of malaria vectors. These data are useful for evaluating the effectiveness of IRS and LLITNs and for long term planning regarding choice of appropriate insecticide.

Mosquito collections from window traps were described previously by Sharp *et al *[2]. Briefly, the contents of window traps were emptied daily by the home owner into pre-labelled specimen jars containing isopropanol. Night control sheets specifying the nights worked were documented and both jars and sheets were collected and replaced at four week intervals. Mosquitoes were collected during an eight month period, from December 2006 to July 2007, before the start of the first spray round to establish a baseline for comparison between pre- and post- intervention periods.

### Identification of vector species, molecular forms, *kdr *and AChE mutations

Mosquitoes were separated into *Culicinae *and *Anophelinae *and counted. Anophelines were morphologically identified into *An. gambiae s.l., An. funestus, Anopheles moucheti *and *Anopheles nili s.l.*, using the keys described by Gillies and De Meillon [6], Gillies and Coetzee [7] and Hervy *et al *[8] and, subsequently, stored in isopropanol.

DNA was extracted from the head and thorax [9] of a sub-sample of mosquitoes to determine family member species using Polymerase Chain Reaction (PCR). The protocols of Scott *et al *[10], Koekemoer *et al *[11], Kengne *et al *[12,13] were used to identify family members of *An. gambiae s.l., An. funestus*, *An. nili s.l*. and *An. moucheti *respectively and the molecular forms of *An.gambiae sensu stricto (s.s.) *were determined according to the method described by Fanello *et al *[14]. The presence of the west-African Leu-Phe and east-African Leu-Ser *kdr *mutations was determined in 505 *An. gambiae s.s *individuals using the TaqMan PCR protocol described by Bass *et al *[15]. A new TaqMan assay devised by Bass *et al *[16] was used to determine *Plasmodium falciparum *sporozoite rates. Insensitive AChE was determined using the PCR method described by Weil *et al *[17].

### Transmission risk

The number of infective mosquitoes per trap per 100 nights for each species complex was calculated as a measure of transmission risk.

### Sequence analysis of *kdr*

The results of *kdr *genotyping using the TaqMan assay were verified by sequencing the relevant region of the sodium channel gene in 50 of the 505 samples analysed. This was carried out using two primers (MosF1, 5'-GATAATGTGGATAGATTCCCCG-3 and MosR1, 5'-CGTTGGTGCAGACAAGGATG-3') flanking the mutation site. PCR reactions (25 μl) contained 1 μl of genomic DNA, 12.5 μl of 2× PCR master mix (Promega) and 100 ng of each primer. PCR products were ethanol precipitated and direct sequenced using an internal primer (MosSeq1 5'-CCATGATCTGCCAAGATGGA-3') and the ABI BigDye Terminator Cycle Sequencing kit followed by analysis on a 310 Automated DNA Sequencer (PE Applied Biosystems).

### Statistical analysis

The genotypic frequencies at the kdr locus were compared to Hardy-Weinberg expectations using the exact test procedures implemented in GenePOP (ver.3.4) software [18].

## Results

### Mosquito collections and molecular identification

A total of 6,162 *Anopheles *mosquitoes were collected of which 4,808 (78%) were morphologically identified as *An. gambiae s.l.*, 120 (2%) *An. funestus*, 1,069 (17%) *An. moucheti *and 165 (3%) *An. nili s.l. *(Table 1). The four identified family groups were found sympatrically in all four provinces. Large variations in mosquito numbers and mosquito species composition existed between sentinel sites and also between different months.

**Table 1:** Total number of anophelines caught per province December 2006–July 2007

Province	*An. gambiae s.l.*	*An. funestus*	*An. moucheti*	*An. nili s.l.*
Centro Sur	225	3	382	26
Litoral	3262	76	36	113
Wele Nzas	580	29	153	23
Kie-Ntem	741	12	498	3

Total	4808	120	1069	165

*Anopheles gambiae s.s. *and *Anopheles melas *were the only two members of the *An. gambiae *complex to be identified (n = 930). *An. gambiae s.s *was identified from 29 of the 30 sentinel sites and accounted for 776 of the *An. gambiae s.l. *identifications. *An. melas *was predominantly identified from the two coastal port cities of Cogo and Mbini where they made up 88% (n = 112) and 79% (n = 67) respectively of the total *An. gambiae s.l. *identified at these two sites. Two specimens were also collected from Yengue, a town in the north-west of Rio Muni, bordering on Cameroon. The S-molecular form of *An. gambiae s.s. *was found in all four provinces and the M-form, only in Litoral, where it accounted for 44% of identifications (n = 350). The two forms were found sympatrically and no hybrids were identified.

*Anopheles moucheti *was the second most abundant vector accounting for 17% of the total and was identified from 22 sites. It was the only member of the *An. moucheti *group to be identified. *An. funestus *was collected from fourteen sentinel sites and was the only member of the *An. funestus *group to be identified and accounted for 2% of the total number of Anopheline mosquitoes caught.

*Anopheles nili s.l. *accounted for 3% of the total *Anopheles *population and was found at seven sentinel sites: Ayamiken, Ayene, Machinda, Ngong, Niefang, Nkue and Yengue. Of the 151 *An. nili s.l. *tested, 98 and 53 were identified as *Anopheles ovengensis *and *Anopheles carnevalei *respectively. *Anopheles carnevalei *was only identified from Yengue where it accounted for 50% of the total *An. nili *s.*l*. identified.

### *Plasmodium falciparum *sporozoite and transmission rates

Sporozoite rates were 4.1% (n = 49) for *An. funestus*, 4.1% (n = 74) for *An. ovengensis*, 3.3% (n = 603) for *An. gambiae s.s. *and 1.6% (n = 126) for *An. moucheti*. The sporozoite rate for *An. melas *was 4.4% (n = 137). *Anopheles carnevalei *was not shown to be involved in transmission although numbers tested were low (n = 52). The estimated number of *An. gambiae s.l., An. moucheti, An. nili s.l. *and *An. funestus *per window trap per 100 nights was 15.5, 3.4, 0.5 and 0.4 respectively. Using the species complex sporozoite prevalence (3.5%, 1.6% 2.6% and 4.1% respectively) the number of infective mosquitoes per trap per 100 nights for each species was 0.5, 0.06, 0.01 and 0.02 respectively.

### *Kdr *allele frequencies in M and S molecular forms of *An. gambiae *s.s

393 S and 113 M molecular forms of *An. gambiae s.s*. were analysed for the presence of *kdr-w *and *kdr-e *alleles using a recently described TaqMan assay (Table 2). The results using the new assay were compared with sequencing in a subset of 50 of the specimens analysed and the two methods were found to be in complete agreement. *Kdr-e *and *kdr-w *resistance alleles were present in S forms with a higher frequency of the *kdr-w *allele (59%) than the *kdr-e *allele (19%). Both alleles also occurred in the M-forms but at much lower frequencies of 9.7% for *kdr-w *and 1.8% for *kdr-e*. Both the *kdr-w *and *kdr-e *alleles were present in S form samples in all four provinces with frequencies of the *kdr-w *allele of 51% in Litoral, 47% in Centro Sur, 64% in Wele Nzas and 73% in Kie Ntem and frequencies of the *kdr-e *allele of 32% in Litoral, 24% in Centro Sur, 8% in Wele Nzas and 14% in Kie Ntem. The *kdr-w *and *kdr-e *alleles were found to co-occur in a single M form specimen and in 103 S form specimens (Table 2). Sample numbers were sufficient to compare *kdr *gene frequencies with Hardy Weinberg expectations in populations collected from a number of sites. These included Bata City in Litoral, Bisun in Centro Sur, Mongomeyen in Wele Nzas and Ebebiyin in Kie-Ntem. Genotypic frequencies of both M and S form populations in Bata City showed significant deviations from Hardy-Weinberg expectations with a heterozygote deficit (P < 0.001). The same was true of the S form population in Bisun in this instance due to a heterozygote excess (P < 0.05). The genotypic frequencies of the S form populations at the other two localities were not significantly different from Hardy Weinberg expectations (P = 1 for the Mongomeyen population and P = 0.39 for the Ebebiyin population).

**Table 2:** Kdr genotype frequencies in *An. gambiae s.s. *in Rio Muni, 2006–2007

Province	District	Locality	n	molecular form	kdr genotypes					
					S/S	S/Rw	S/Re	Rw/Rw	Re/Re	Re/Rw
Litoral	Bata	Yengue	25	M	20	4	1	0	0	0
			33	S	3	3	5	10	2	10
		Ayamiken	5	S	1	3	1	0	0	0
		Machinda	1	S	0	1	0	0	0	0
	Bata City	Ngolo	9	M	6	0	0	1	1	1
			29	S	1	0	0	11	6	11
		Etofili-Lubi	1	M	1	0	0	0	0	0
			9	S	0	0	0	6	1	2
		Centro	2	S	0	0	0	0	0	2
		Ukomba	60	M	51	9	0	0	0	0
			12	S	4	1	0	1	1	5
		Ncolombong	11	M	8	2	0	1	0	0
			19	S	1	0	0	2	2	14
	Cogo	Mbini	6	M	4	1	0	0	0	0
			5	S	1	4	0	1	0	0
		Cogo	1	M	1	0	0	0	0	0
			1	S	0	0	0	1	0	0
Centro Sur	Niefang	Niefang	13	S	0	3	2	2	1	5
		Bisun	48	S	3	20	5	6	2	12
	Evinayong	Bicurga	5	S	2	2	0	1	0	0
		Evinayong	17	S	2	3	1	7	0	4
	Akurenam	Akurenam	5	S	1	0	0	1	0	3
Wele Nzas	Anisok	Ayene	23	S	2	11	1	4	0	5
		Anisok	3	S	0	0	0	2	0	1
	Mongomo	Mongomeyen	49	S	4	19	2	19	0	5
		Mongomo	4	S	0	0	0	4	0	0
		Asok	1	S	0	1	0	0	0	0
	Nsork	Nsork	11	S	1	2	0	7	0	1
		Aconibe	6	S	0	1	1	3	0	1
Kie-Ntem	Micomiseng	Nkue	17	S	2	9	2	2	0	2
	Ebebiyin	Ngong	26	S	0	6	1	19	0	0
		Ebebiyin	49	S	1	0	0	29	1	18

### AChE resistance

All 200 mosquitoes tested for insensitive AChE were found to be susceptible.

## Discussion

*Anopheles gambiae s.l. *was shown to be the main vector within this geographical region with the other three species playing a relatively minor role due to their low densities. A sporozoite rate of 4.4% for *An. melas *indicates its involvement in malaria transmission in the two sentinel sites from which it was identified.

Previous studies in Equatorial Guinea have shown *An. gambiae s.l. *and *An. funestus *to be the main vectors of malaria [3-5]. Elsewhere in West and Central Africa *An. gambiae s.l., An. funestus, An. moucheti *and *An. nili s.l*. have been shown to be effective vectors with EIR rates ranging from 1–1000 infective bites per year recorded [19]. In this study, all four identified groups from Rio Muni have been shown to be involved in transmission of malaria with *An. gambiae s.s. *being the major vector. Although *An. funestus *was found to have the highest sporozoite rate, the number caught was very low hence it was not shown to be a major vector.

*Anopheles nili *has recently been described as a complex consisting of four member species based on morphological criteria: *An. nili, Anopheles somalicus, An. carnevalei *and *An. ovengensis *[12].*Anopheles carnevalei *is relatively rare in occurrence and has so far only been reported from the equatorial forests of Ivory Coast and Cameroon [20] and from a village in Equatorial Guinea, Yengue [21,22]. *Anopheles ovengensis *has been reported from southern Cameroon [23]. The results of this study further provide proof of the distribution of *An. ovengensis *to extend throughout the northern part of Equatorial Guinea as was suggested by Awono-Ambene [23] and confirms the presence of *An. carnevalei *in Yengue, a village in the north-west of the mainland where it was sympatric with *An. ovengensis*. These collections were all made from window traps thus indicating some degree of endophilic behaviour although previous studies suggest predominately exophilic habits [23].

Resistance of mosquitoes to insecticides usually arises through one of two mechanisms, or a combination of the two; metabolic resistance due to increased production of detoxifying enzymes and target site resistance due to mutations in the sodium channel, acetylcholinesterase or GABA receptor [24]. *Kdr *is a target site resistance of the sodium channel and is one of the mechanisms conferring resistance to pyrethroid and DDT insecticides. Two mutations have been described, a leucine-phenylalanine substitution originally found in west-African *An. gambiae s.l*. [25] and a leucine-serine substitution found in east-African *An. gambiae s.l*. [26]. However, recent studies in Cameroon and Gabon have shown that these mutations are not unique to these geographical regions and that there is considerable overlap with both being present in the same populations [27,28]. Both the resistance alleles were identified in the populations examined in this study. The *kdr-w *and *kdr-e *alleles were present at low frequencies in M forms (9.7% and 1.8%) and in much higher frequencies in S forms with the frequency of the *kdr-w *allele 59% and the frequency of the *kdr-e *allele 19%. The observed gene frequencies are in close concordance with those reported recently in the neighbouring country of Cameroon where *kdr-w *and *kdr-e *alleles were present in M form populations at frequencies of 6.3% for *kdr-w *and 1.1% for *kdr-e *and in S form populations at frequencies of 52.7 and 13.9% [29]. This correlation in observed gene frequencies could indicate that the *kdr *alleles have migrated from Cameroon to Equatorial Guinea or vice versa. It would be interesting in future to sequence the sodium channel gene regions flanking the *kdr *locus, in particular intron I upstream of the mutation site, as this will provide evidence as to whether the *kdr *mutations have arisen once and spread between the two countries or represent independent mutation events. It may also reveal the extent of migration between populations in Equatorial Guinea and Cameroon. Interestingly the gene frequencies we observed on the mainland differ significantly from those seen on the island part of Equatorial Guinea, Bioko, where prior to the onset of the spray programme, 50% of the M-forms carried the *kdr-w *allele in either the homozygous or heterozygous form while it was completely absent in the S-form [30,2]. However, previous studies have suggested that *An. gambiae *populations on Bioko are to a large extent isolated from mainland populations [29].

As reported previously in the neighbouring countries of Cameroon and Gabon [28,29] we observed a large number of Re/Rw genotypes in the localities sampled in this study (26% of S form mosquitoes carried this genotype). Indeed this was the predominant genotype seen in S form mosquitoes after the Rw/Rw genotype (34%). A recent study has shown that this genotype confers a significant degree of resistance to DDT, although the level of resistance is not significantly greater than that conferred by Rw/Rw [29]. Significantly we also recorded this genotype in a single M form specimen. This result was confirmed by sequencing and to our knowledge represents the first report of this genotype in M form mosquitoes. Further screening for *kdr *in M form populations in Equatorial Guinea will reveal the extent of this genotype in the M form but this initial study indicates it may be currently found at an extremely low frequency.

In the Bata City area of Litoral both M and S populations showed significant deviations from Hardy-Weinberg expectations (P < 0.001) and this was due to a heterozygote deficit. As *kdr *is a recessive trait [25,26] and only homozygous genotypes express the resistance phenotype, studies need to be implemented to determine the origin of the insecticide selection pressure as is observed from the high frequency of homozygous resistant individuals in Bata City before IRS. Clearly the presence of both *kdr-e *and *kdr-w *alleles at high frequencies in these populations may have implications for the effectiveness of the current vector control programme which is based on pyrethroid insecticides.

AChE is the target site of organophosphates and carbamate insecticides and insensitive AChE in mosquitoes coincides with high insecticide resistance to these insecticide classes. No insensitive AChE was detected in this baseline study indicating continued efficacy of these insecticide classes. Coleman *et al *[31] reviewed published insecticide resistance data in Africa and found eight sites with reported carbamate resistance and 13 sights with organophosphate resistance. They attributed this to the limited application of carbamates and organophophates in large-scale vector control and the lack of resistance monitoring.

This study provides contemporary information on the distribution of malaria species and their role in malaria transmission in Rio Muni, Equatorial Guinea. It also provides useful information on measures of insecticide resistance for the vector control programme. Pyrethroids have been selected as the insecticide of choice for the first spray round due to its low toxicity in humans, its longer residual effect and for cost efficacy and procurement implications. WHO susceptibility tests in 2000 showed resistance to DDT but susceptibility to both deltamethrin and permethrin [5,32]. In bioassays conducted in 2007 with alphacypermethrin (Fendona), there was 95.5% mortality in the exposed group by the end of the observation period (24 hrs) (personnel communication M. Torrez 2007). In West Africa, large scale agricultural pyrethroid use has resulted in very high insecticide resistance [3]. However in Equatorial Guinea pyrethroids have not been widely used as an agricultural insecticide. Therefore this studies findings of *kdr *mutations at such high frequency in mosquito populations in Equatorial Guinea (particularly in S form populations) is unexpected. Nevertheless, the presence of *kdr *alleles at the observed frequencies could impact on the choice of insecticide for future spray rounds and will require ongoing monitoring and evaluation to ensure the chosen insecticide remains effective, a process the EGMCI has put in place. Carbamates remain a viable alternative in the absence of insensitive AChE and have been successfully used in a number of spray programmes including on the island of Bioko and in Mozambique [2,33]. Further biochemical testing is planned to determine whether or not other resistant mechanisms are present in the mosquito populations and to assess if these develop as a result of selection pressure exerted by the IRS and ITN vector control operations.

## Competing interests

The authors declare that they have no competing interests.

## Authors' contributions

FCR co-designed the study, carried out laboratory analyses of mosquitoes, participated in data analysis and interpretation and was involved in the drafting of the manuscript. CB carried out *kdr *laboratory analyses, analysed and interpreted the *kdr *results and assisted in the drafting of the manuscript. MT carried out the susceptibility assays and contributed to the drafting of the manuscript. DG managed the database, assisted with the analysis of results and contributed to the manuscript. VR assisted with laboratory analyses and helped draft the manuscript. LY was responsible for the IRS programme, monitoring of window traps and helped draft the manuscript. AE was responsible for the window trap collections and preparation of mosquitoes for analysis and helped draft the manuscript. CS was responsible for the overall management of the control programme and assisted in drafting the manuscript. PM assisted with mosquito collections and helped draft the manuscript. RM helped draft the manuscript and critical evaluation thereof. IK co-designed and coordinated the study and was involved in the drafting of the manuscript and critical evaluation thereof. All authors read and approved the manuscript.
